# Systematic review of ocular and circulatory cytokines linking diabetic retinopathy to kidney disease

**DOI:** 10.3389/fendo.2026.1767086

**Published:** 2026-03-11

**Authors:** Yuyan Tang, Zhen Li, Ziyu Jia

**Affiliations:** 1 College of Optometry and Ophthalmology, North Sichuan Medical College, Sichuan, China; 2 Department of Ophthalmology, Clinical Medical College & Affiliated Hospital of Chengdu University, Sichuan, China; 3 Department of Ophthalmology, The People’s Hospital of Leshan, Sichuan, China

**Keywords:** diabetic retinopathy, diabetic kidney disease, cytokines, vascular endothelial growth factor, inflammation

## Abstract

**Background:**

Diabetic retinopathy (DR) and diabetic kidney disease (DKD) are major microvascular complications of diabetes that contribute substantially to visual disability and renal failure worldwide. Increasing evidence supports the concept of an “eye–kidney axis,” whereby inflammatory and angiogenic dysregulation may drive parallel microvascular injury in the retina and kidney. The present systematic review aimed to synthesize current clinical evidence on the association between vitreous and serum cytokines and renal impairment in patients with DR.

**Methods:**

Following PRISMA guidelines, PubMed, Embase, and Web of Science were systematically searched (2005–2025). We included observational human studies investigating vitreous or serum cytokines in adult patients with clinically staged DR and their associations with renal function parameters, including estimated glomerular filtration rate (eGFR) and albuminuria. Data on study characteristics, cytokine profiles, renal metrics, and major findings were extracted. Study quality was assessed using the modified Newcastle–Ottawa Scale. Due to heterogeneity in study design and outcomes, results were synthesized narratively.

**Results:**

Seventeen eligible studies involving patients with type 1 and type 2 diabetes were included. Key cytokines assessed included angiogenic factors (VEGF, PlGF, ANGPTL2/4) and inflammatory mediators (TNF-α, IL-6, IL-17A, MCP-1, sRAGE), measured predominantly using ELISA. Across studies, both vitreous and serum VEGF levels were consistently elevated in proliferative DR and showed significant associations with albuminuria and reduced eGFR. Pro-inflammatory cytokines including TNF-α, IL-17A, and progranulin correlated with DR severity and markers of DKD progression. Biomarkers such as s(P)RR, FABP4, and Ephrin-A1 also exhibited concordant trends with microvascular dysfunction in both organs. Overall, the compiled evidence indicates that inflammatory activation and aberrant angiogenesis are common molecular signatures linking retinal and renal microangiopathy.

**Conclusions:**

This systematic review supports the hypothesis that DR and DKD share overlapping inflammatory and angiogenic pathways, reflected by synchronized changes in vitreous and systemic cytokine levels. These findings highlight the potential of retinal-derived biomarkers for non-invasive risk stratification of DKD, and suggest therapeutic value in combined anti-inflammatory and anti-angiogenic strategies to protect eye–kidney microvascular integrity. High-quality longitudinal and mechanistic studies are warranted to further establish causality within the eye–kidney axis and translate biomarker-based surveillance into clinical practice.

## 1 Introduction

Diabetic microvascular complications, including diabetic retinopathy (DR) and diabetic kidney disease (DKD), pose significant challenges to global healthcare systems. These conditions are not only highly prevalent but also among the most severe complications of diabetes, contributing to increased disability and mortality rates. Epidemiological studies show that approximately 20%-50% of patients with type 2 diabetes will eventually develop DKD (1), while the overall prevalence of DR in individuals with diabetes is approximately 22% (2). As these complications frequently coexist, they exacerbate morbidity in diabetic patients.

DR results from chronic hyperglycemia-induced damage to the retinal microvasculature, leading to microvascular dilation, leakage, and neovascularization, ultimately causing vision loss. DR is diagnosed primarily through non-invasive fundus examination, which reveals retinal hemorrhages, hard exudates, and neovascularization (2). DR stages range from non-proliferative diabetic retinopathy (NPDR) to proliferative diabetic retinopathy (PDR), with the latter strongly associated with severe vision impairment.

DKD is a common microvascular complication in diabetic patients, typically characterized by proteinuria and a progressive decline in renal function. The pathology of DKD primarily involves damage to both the renal tubules and glomeruli, including glomerulosclerosis, tubulointerstitial fibrosis, and capillary basement membrane thickening (1). DKD is diagnosed based on urinary albumin-to-creatinine ratio (UACR) and estimated glomerular filtration rate (eGFR), although renal biopsy, the gold standard for diagnosis, is rarely performed due to its invasive nature.

Both DR and DKD share common pathogenic mechanisms, such as endothelial dysfunction, oxidative stress, chronic inflammation, and dysregulated angiogenesis, all of which are aggravated by hyperglycemia (3). This overlap has led to the development of the “eye–kidney axis” concept (4), which posits that diabetes-induced microvascular damage is reflected in both the eye and kidney. These organs are interconnected through shared inflammatory and angiogenic pathways, with key mediators such as vascular endothelial growth factor (VEGF), tumor necrosis factor-α (TNF-α), interleukin-6 (IL-6), and monocyte chemoattractant protein-1 (MCP-1), contributing to both retinal neovascularization and glomerular injury (5, 6).

The eye–kidney axis concept extends beyond local interactions between these two organs to encompass systemic processes, such as chronic inflammation and oxidative stress, which exacerbate endothelial dysfunction, increase microvascular damage, enhance vascular permeability, and promote neovascularization in both the retina and kidneys. Ultimately, these factors lead to structural and functional impairments in both organs (4).

Given the eye’s unique anatomical structure and transparency, it serves as an ideal “window” for assessing systemic vascular damage. Fundus examination offers a non-invasive means of diagnosing DR and can also indirectly reflect changes in the renal microvasculature, providing valuable insights into DKD. Early screening of both ocular and renal biomarkers is critical for timely intervention, improving disease management, and preventing irreversible damage.

Vitreous fluid, obtainable during vitrectomy, reflects the local retinal microenvironment, whereas serum and plasma samples provide insight into systemic inflammation and vascular impairment. Thus, defining the relationships between vitreous and circulating cytokines (e.g., VEGF, TNF-α, IL-6) and renal function parameters may deepen understanding of the eye–kidney axis and facilitate early risk stratification through blood-based biomarkers, potentially in combination with imaging or liquid-biopsy approaches.

Although numerous clinical studies have examined the association between DR and DKD, most are single-center, cross-sectional, or case-control in design, with inconsistent findings and limited longitudinal validation. Therefore, this systematic review aims to synthesize current evidence, evaluate concordance between retinal and systemic microvascular alterations, and identify gaps for future research, ultimately supporting the clinical translation of the eye–kidney axis concept.

## 2 Methods

### 2.1 Search strategy

This systematic review was conducted in accordance with the PRISMA 2020 guidelines. PubMed, Embase, and Web of Science were systematically searched for English-language articles published from January 2005 to January 2025. Eligible studies investigated associations between vitreous and/or serum cytokine levels and renal function parameters (e.g., eGFR, UACR) or DKD severity in adult patients with diabetic retinopathy (DR). The search strategy incorporated both controlled vocabulary (MeSH for PubMed; Emtree for Embase) and free-text keywords, including synonyms and spelling variants. Boolean operators and database-specific adaptations were applied.

The PubMed search string was as follows: [(vitreous OR vitreous body OR serum) AND (cytokines OR VEGF OR vascular endothelial growth factor A OR TNF-α OR tumor necrosis factor-α)] AND (diabetic retinopathy OR DR) AND (diabetic nephropathy OR diabetic kidney disease OR eGFR OR albuminuria OR UACR OR ACR OR creatinine).

### 2.2 Inclusion criteria (PICO framework)

Population (P): Adult patients with type 1 or type 2 diabetes mellitus with clinically documented DR staging. Presence or absence of DKD was permitted.

Intervention/Exposure (I): Quantification of vitreous or serum cytokines, including but not limited to VEGF, TNF-α, IL-6, IL-8, and MCP-1/CCL2.

Comparator (C): Comparisons across DR stages, DKD risk categories, or renal functional status.

Outcome (O): Associations between cytokine levels and renal function; differences in cytokine expression stratified by DKD severity; biomarker diagnostic or prognostic performance.

Study Design: Observational cohort or case-control studies, including those obtaining vitreous samples during ophthalmic procedures.

### 2.3 Exclusion criteria

Studies were excluded if they met any of the following criteria (1): non-diabetic retinal diseases or concomitant ocular inflammatory conditions (2); receipt of intraocular anti-VEGF therapy within the previous 3 months (3); non-human, *in vitro* studies, or single case reports (n = 1) (4); absence of renal function data (5); review articles, conference abstracts, editorials, or mechanistic commentaries.

### 2.4 Data extraction and synthesis

For each included study, data were extracted on: author, publication year, country, study design, sample size, DR stage (PDR or NPDR), specimen type (vitreous fluid, serum), laboratory assay, cytokines measured, renal parameters (eGFR, UACR/ACR, serum creatinine), and principal results. Because of substantial heterogeneity in measurement methods and outcome reporting, a narrative synthesis was conducted rather than a quantitative meta-analysis.

### 2.5 Quality assessment

Methodological quality was evaluated using the modified Newcastle–Ottawa Scale (NOS), including selection (up to 4 points), comparability (up to 2 points), and exposure/outcome assessment (up to 3 points), with total scores ranging from 0 to 9. Two reviewers independently assessed each study, resolving disagreements through discussion or third-reviewer adjudication. Selection bias, measurement bias, and confounding were specifically evaluated to ensure reliability and interpretability of the findings (**Table 1**).

**Table 1:** Risk of bias assessment of the 17 included studies using the modified Newcastle–Ottawa Scale (NOS).

Study	Design	Selection (0–4)	Comparability (0–2)	Outcome (0–3)	Total (0–9)	Quality Level	Justification
Lampropoulou et al., 2014 (1)	Cross-sectional observational study	3	1	2	6	Fair	No confirmed consecutive enrollment; metabolic imbalance with limited adjustment; assays lacked blinding and batch control
Mao D. et al., 2020 (7)	Cross-sectional observational study	2	1	1	4	Poor	Single-center, small convenience sample with limited generalizability and residual baseline confounding; DR diagnosis and ELISA were reliable, but the cross-sectional design precluded longitudinal outcome assessmen
Liu J. et al., 2022 (8)	Retrospective (*post hoc*) cohort	3	1	2	6	Fair	Single-center cohort with potential selection bias; partial adjustment for confounders; surgical outcomes were clearly defined but follow-up completeness was not fully ensured.
Chen X.et al., 2023 (9)	Cross-sectional observational	2	1	2	5	Fair–Poor	Highly limited representativeness with incomplete confounder control; although measurements were objective, the cross-sectional design lacked temporal assessment.
Mahdy R.et al., 2011 (10)	Cross-sectional observational	2	1	2	5	Fair–Poor	Single-center study with limited representativeness and partial confounder control; VEGF measurements were objective but the cross-sectional design prevented temporal or causal inference.
Wu G.et al., 2021 (11)	Cross-sectional observational	2	1	2	5	Fair–Poor	Single-center cross-sectional cohort with limited representativeness and partial confounder adjustment; vitreous factor measurements were objective, but temporal or causal inference was not possible.
Itoh K.et al., 2021 (12)	Cross-sectional observational	2	1	2	5	Fair–Poor	Single-center cross-sectional cohort with limited representativeness and partial confounder control; vitreous FABP4 measurement was objective, but temporal or causal inference was not possible.
Mathala N.et al., 2019 (13)	Cross-sectional observational	2	1	2	5	Fair–Poor	Single-center cross-sectional cohort with limited representativeness and partial confounder control; biomarker measurements were objective but causal inference was not possible.
Baharivand N.et al., 2012 (14)	Case-control observational	2	1	2	5	Fair–Poor	Single-center cross-sectional cohort with limited representativeness and partial confounder control; VEGF measurements were objective but causal inference was not possible.
Katagiri et al., 2017 (15)	Cross-sectional observational	2	1	2	5	Fair–Poor	Single-center cross-sectional cohort with limited representativeness and partial confounder control; vitreous sRAGE measurement was objective but lacked temporal or causal inference
Quevedo-Martínez J. U., et al., 2021 (16)	Cross-sectional observational	2	1	2	5	Fair–Poor	Single-center cross-sectional cohort with limited representativeness and incomplete confounder control; serum cytokine measurements were objective but lacked temporal or causal inference.
Xu L., et al., 2015 (17)	Cross-sectional observational	2	1	2	5	Fair–Poor	Single-center cross-sectional cohort with limited representativeness; serum progranulin was measured objectively, but causal inference was not possible.
Wang Wensu., et al., 2025 (18)	Cross-sectional observational	2	1	2	5	Fair–Poor	Single-center cross-sectional cohort with limited representativeness; serum IL-17A was measured objectively, but causal inference was not possible.
Hase K., et al., 2017 (19)	Cross-sectional observational	2	1	2	5	Fair–Poor	Single-center cross-sectional cohort with limited representativeness; plasma s(P)RR was measured objectively, but causal inference was not possible.
Klein B. E. K. et al., 2012 (20)	Cross-sectional observational	2	1	2	5	Fair–Poor	Single-center cross-sectional cohort with limited representativeness; blood and clotting factor measurements were objective, but causal inference was not possible.
Hamid et al., 2022	Cross-sectional observational	2	1	2	5	Fair–Poor	Single-center cross-sectional cohort with limited representativeness; urine and serum VEGF were measured objectively, but causal inference was not possible.
Hanefeld et al., 2016 (21)	Cross-sectional observational	2	1	2	5	Fair–Poor	Single-center cross-sectional cohort with limited representativeness; VEGF was measured objectively, but causal inference was not possible.

## 3 Results

### 3.1 Study selection

**Figure 1:**
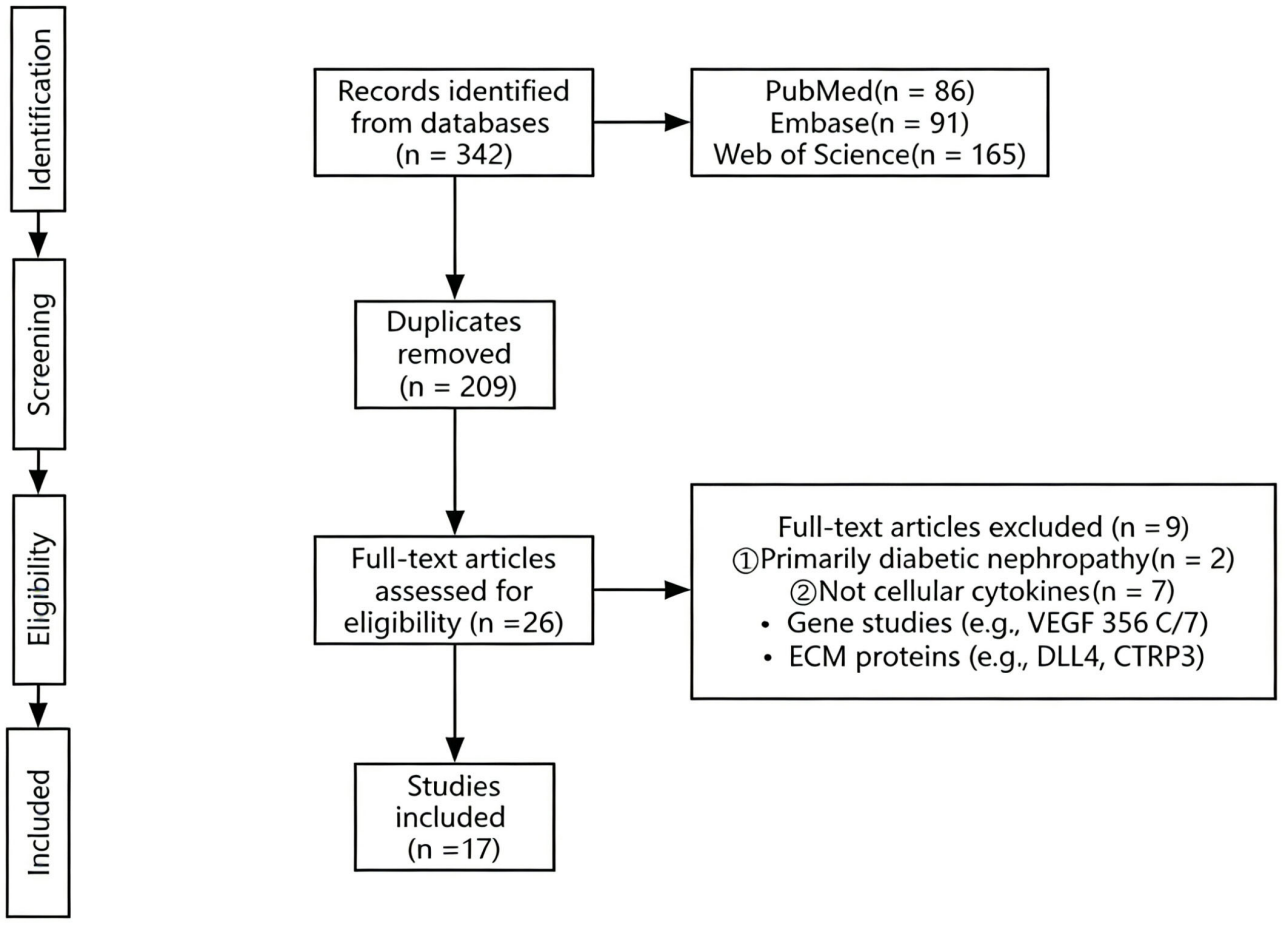
Risk of bias assessment of the 17 included studies using the modified Newcastle–Ottawa Scale (NOS).

A total of 342 records were identified through searches of PubMed, Embase, and Web of Science. After removing duplicates, 209 articles remained for title and abstract screening. Following initial screening, 26 full-text articles were assessed for eligibility, of which 8 were excluded for not meeting the inclusion criteria. Ultimately, 17 observational cohort studies were included in the systematic review (**Figure 1**, PRISMA flow diagram).

### 3.2 Study characteristics

Seventeen clinical observational studies were included, involving adult patients with type 1 or type 2 diabetes across varying stages of DR (no DR, NPDR, and PDR) and different DKD risk categories. Sample sizes ranged from approximately 20 vitreous samples to over 400 serum samples. Biomarkers assessed primarily comprised angiogenic factors (e.g., VEGF, PlGF, ANGPTL4, ANGPTL2), inflammatory cytokines (e.g., TNF-α, IL-6, IL-17A, sRAGE, soluble (pro)renin receptor [s(P)RR]), and extracellular matrix–related proteins (e.g., syndecan-1). Most studies utilized ELISA, whereas a subset used multiplex immunoassays. Renal function was evaluated using eGFR, serum creatinine (sCr), blood urea nitrogen (BUN), and albuminuria metrics (UACR/ACR) (**Table 2**).

**Table 2:** Characteristics of included studies evaluating vitreous/serum inflammatory and angiogenic cytokines in diabetic retinopathy and their associations with diabetic kidney disease.

Study (year)	Country	Design	Sample size	DR stage (n)	sampled	Cytokines/Factors	Assay	DKD metrics reported	Main Findings
Lampropoulou et al., 2014 (22)	Greece	Cross-sectional observational	80	31DR	Serum, urine	TNF-α	ELISA	ACR, eGFR	Urinary TNF-α levels showed a strong positive association with albumin-to-creatinine ratio (ACR), whereas serum TNF-α demonstrated no significant correlation with ACR
Mao D. et al., 2020 (7)	China (Ningbo, Zhejiang)	Cross-sectional observational	55	20DR VS 16DM VS 19non-DM	Plasma	Ephrin-A1,VEGF_165_	ELISA	sCr	Urinary TNF-α exhibited a strong positive correlation with ACR, while serum TNF-α showed no significant relationship.
Liu J. et al., 2022 (8)	China	Retrospective (*post hoc*) cohort	45	45 PDR	Vitreous fluid, aqueous humor, serum	VEGF-A	Cytometric Bead Array	eGFR	VEGF-A levels in vitreous, aqueous humor, and serum did not differ across renal function groups, indicating that kidney function does not parallel PDR severity.
Chen X.et al., 2023 (9)	China (Beijing)	Cross-sectional observational	122	23DM vs 35DR vs 64DR + DKD	Plasma	MCP-1, IL-6, IL-8, VEGF-A, VEGF-C, VEGF-D, PlGF	Protein microarrays	uACR, eGFR, sCr	LDL-C and VEGF-D are independent risk factors for DR; circulating PlGF and VEGF-D positively correlate with uACR grade;
Mahdy R.et al., 2011 (10)	Egypt (Zagazig)	Cross-sectional observational	50	10 no complications vs 10 NPDR vs 10 PDR vs 10 non-DM	serum	VEGF	ELISA	24 h urine albumin, creatinine,	Serum VEGF levels were significantly higher in PDR patients compared with the NPDR group, concomitant with elevated urinary albumin levels.
Wu G.et al., 2021 (11)	China	Cross-sectional observational	75	38 PDR vs 37 non-DM	Vitreous fluid	Syndecan-1, PIGF, ANGPTL-4, VEGF, IL-6, IL-8, MCP-1, TNF-α, ICAM-1	Multiplex immunoassay, Cytometric bead array	sCr,BUN,eGFR	Vitreous syndecan- 1, PIGF, ANGPTL- 4, VEGF, and IL-8 were elevated in PDR; non-VEGF factors correlated positively with sCr/BUN and negatively with eGFR, linking ocular inflammation/angiogenesis to renal dysfunction.
Itoh K.et al., 2021 (12)	Japan	Cross-sectional observational	40	20PDR vs 20Non-PDR	Vitreous fluid	FABP4, VEGF-A	ELISA	sCr, BUN, eGFR	Vitreous FABP4 significantly higher in PDR than non-PDR; FABP4 positively correlates with scr
Mathala N.et al., 2019 (13)	India	Cross-sectional observational	150	50 non-DM vs 50 NPDR vs 50 PDR	Serum	TNF-α, VEGF	ELISA	sCr	sCr, TNF-α and VEGF were significantly higher in diabetic patients with retinopathy vs those without
Baharivand N.et al., 2012 (14)	Iran	Case-control observational	65	30 NPDR vs 30 PDR	Vitreous fluid, Serum	VEGF-A	ELISA	ACR, sCr	Vitreous and serum VEGF levels were higher in PDR vs NPDR; serum VEGF positively correlated with ACR, and VEGF was significantly lower in early nephropathy stage.
Katagiri et al., 2017 (15)	Japan	Cross-sectional observational	33	8 NPDR vs 23 PDR	Vitreous fluid	sRAGE, VEGF	ELISA	eGFR, sCr	Higher vitreous sRAGE was associated with worse renal function, showing a positive correlation with serum creatinine and an inverse correlation with eGFR
Quevedo-Martínez J. U. et al., 2021 (16)	Mexico	Cross-sectional observational	64	16 non-DM vs 16 no DR vs 16 NPDR vs 16 PDR	Serum	IL-1β, IL-2, IL-4, IL-6, IL-8, IL-10, IL-12, IL-17A, TNF-α, IFN-γ	Cytometric Bead Array	sCr	IL-6 and TNF-α correlated positively with serum creatinine, suggesting a link between inflammation and DR severity.
Xu L. et al., 2015 (17)	China	Cross-sectional observational	96	84 T2DM vs 12 non-DM	Serum	PGRN, IL-6, TNF-	ELISA	UACR, sCr, BUN, eGFR	PGRN increased with severity, positively correlated with UAER and creatinine, negatively correlated with eGFR; UAER and creatinine were independent predictors of PGRN
Wang W. et al., 2025 (18)	China	Cross-sectional observational	138	54 no DR vs 61 DR	Serum	IL-17A	Ultra-sensitive high-sensitivity immunoassay	sCr, eGFR, ACR	Serum IL-17A was significantly higher in DKD patients; IL-17A positively correlated with serum creatinine and ACR, negatively with eGFR.
Hase K., et al., 2017 (19)	Japan	Cross-sectional observational	40	20 non-DM vs 20 PDR	Plasma	s(P)RR, prorenin, activated prorenin, TNF-α, CFD, LRG1, VAP-1	ELISA, Multiplex bead-based immunoassay	sCr, eGFR	s(P)RR strongly correlated with TNF-α, CFD and LRG1, and correlated positively with serum creatinine and negatively with eGFR
Klein BE. et al., 2012 (20)	USA (Wisconsin)	Cross-sectional observational	442	216 PDR	serum, plasma	IL-6, von Willebrand factor	ELISA, coagulation assay	Proteinuria, eGFR	nephropathy (proteinuria or low eGFR) showed strong association with both PDR and ME
Hamid et al., 2022	Malaysia	Cross-sectional observational	79	36DN with DR vs 43DN	Serum, urine	VEGF-A	ELISA	creatinine, urine protein-creatinine index	Serum and urine VEGF levels were higher in DN patients with DR, indicating more severe microvascular injury.
Hanefeld et al., 2016 (21)	Germany	Cross-sectional observational	401	302 T2DM vs 99 controls	Serum	VEGF-A	ELISA	UACR	Serum VEGF-A is elevated in T2DM and associated with DKD indicators.

### 3.3 Vitreous cytokines and renal function

Patients with PDR consistently exhibited elevated vitreous levels of VEGF, sRAGE, and FABP4, which showed positive correlations with albuminuria severity and reduced eGFR [Baharivand 2012 (14); Wu 2021 (11); Itoh 2021 (12); Katagiri 2017 (15)]. These findings indicate that local retinal inflammatory and angiogenic dysregulation may reflect or contribute to systemic microvascular damage.

### 3.4 Serum cytokines and renal function

Serum markers including TNF-α, IL-17A, progranulin, and Ephrin-A1 were significantly increased in patients with advanced DR and concomitant renal impairment [Lampropoulou 2014 (22); Mao 2020 (7); Xu 2015 (17); Wang 2025 (18); Hanefeld 2016 (21)]. Notably, inflammatory cytokine levels often worsened in parallel with rising UACR and declining eGFR, suggesting clinical value in DKD risk stratification.

### 3.5 Integration of local and systemic biomarkers

A subset of studies simultaneously measured vitreous and serum cytokines, demonstrating moderate correlations between the two compartments [Chen 2023 (9); Liu 2022 (3); Hase 2017 (19); Klein 2012 (20)]. These results reinforce the proposed “eye–kidney axis,” wherein shared inflammatory and angiogenic pathways contribute concurrently to retinal neovascularization and glomerular injury.

## 4 Discussion

### 4.1 Clinical significance and principal findings

This systematic review integrated 17 clinical observational studies assessing inflammatory and angiogenic mediators in vitreous and serum samples from patients with diabetic retinopathy (DR) and coexisting diabetic kidney disease (DKD), including both T1DM and T2DM populations across different stages of DR and DKD risk. The evidence consistently supports the presence of an ocular–renal microvascular axis, suggesting that retinal pathology reflects systemic microvascular injury.

Systemic inflammatory mediators, including TNF-α, IL-17A, and progranulin, were elevated in DR and significantly correlated with renal dysfunction and microalbuminuria (16, 17, 22, 23). Locally elevated vitreous and serum markers such as VEGF, FABP4, sRAGE, and Ephrin-A1 were associated with advanced DR (particularly PDR), reduced eGFR, and increased albuminuria (8, 10–15, 18, 21). Several studies also reported stage-dependent gradients in circulating biomarkers, reinforcing their potential utility in monitoring disease progression (13). Furthermore, systemic factors such as the soluble (pro)renin receptor and coagulation-related mediators were associated with both retinal microvascular damage and declining renal function (19, 20), highlighting the contribution of systemic inflammation to DR-DKD comorbidity.

Taken together, both vitreous and serum cytokines involved in angiogenesis and inflammation—particularly VEGF, TNF-α, IL-6, IL-17A, sRAGE, FABP4, Ephrin-A1, and progranulin—are elevated in patients with advanced DR and DKD. These findings suggest a positive feedback loop in which inflammatory and angiogenic pathways exacerbate both retinal neovascularization and glomerular injury. Clinically, coordinated evaluation of ocular and systemic biomarkers may support early detection, risk stratification, and therapeutic decision-making. Moreover, anti-inflammatory and anti-angiogenic treatment strategies may jointly benefit ocular and renal microvasculature.

In summary, elevated vitreous and serum inflammatory and angiogenic mediators are closely associated with DR severity and DKD progression, reinforcing the ocular–renal microvascular axis concept. The evidence supports the integration of coordinated ocular and systemic biomarker evaluation to improve early detection, microvascular risk stratification, and integrated management of DR–DKD comorbidity. These findings advocate for the development of integrated, biomarker-based management strategies in diabetes.

### 4.2 Shared inflammatory, angiogenic, and fibrotic pathways in DR and DKD

**Figure 2:**
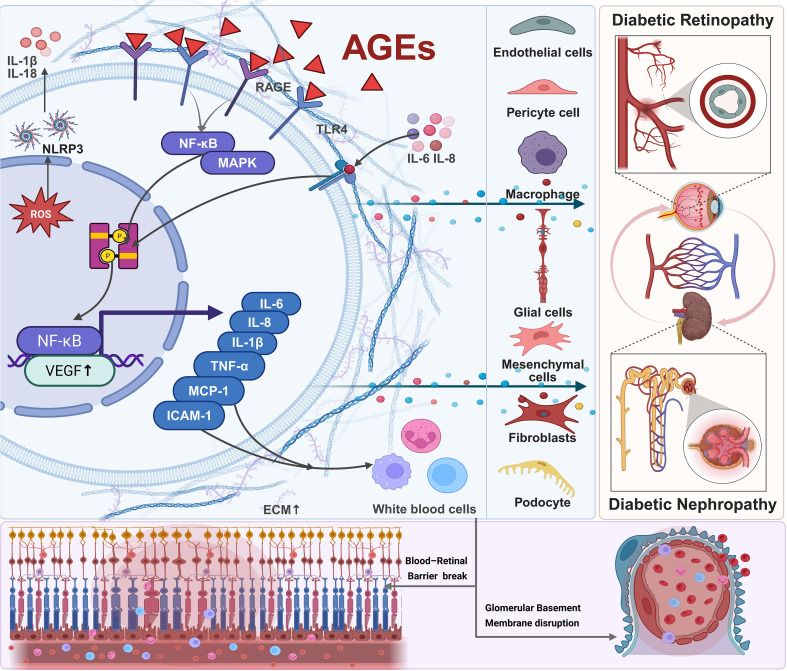
This figure depicts the shared microvascular injury pathways of the eye–kidney axis. Chronic hyperglycemia induces oxidative stress and endothelial dysfunction, activating inflammatory cytokines (e.g., TNF-α, IL-6), angiogenic mediators (e.g., VEGF), and fibrotic regulators (e.g., TGF-β). These processes disrupt the blood–retinal and glomerular filtration barriers, increase vascular permeability, and drive extracellular matrix remodeling and fibrosis. The parallel activation of these pathways in the retina and kidney underlies the cross-organ correlations in biomarker levels and disease severity.

The retina and glomerulus share structural and functional features—both rely on dense microvascular networks and intact endothelial–pericyte or endothelial–podocyte interactions. They are similarly susceptible to hyperglycemia-induced oxidative stress, metabolic dysregulation, and chronic inflammation (24). A conceptual schematic depicting these shared pathogenic pathways is presented in **Figure 2**.

Hyperglycemia promotes the accumulation of advanced glycation end products (AGEs) and mitochondrial reactive oxygen species (ROS), which activate RAGE, TLR4, and NF-κB signaling, increasing inflammatory cytokines such as TNF-α, IL-6, and IL-1β; upregulating adhesion molecules; and enhancing vascular permeability (3). Metabolic stress also activates the NLRP3 inflammasome, facilitating caspase-1-dependent IL-1β and IL-18 maturation, contributing to retinal microglial activation, blood–retinal barrier breakdown, podocyte apoptosis, and glomerulosclerosis (25).

Retinal pericytes and renal podocytes show similar vulnerability to inflammatory insults. Damage or loss of these cells disrupts microvascular stability, leading to retinal microaneurysms and capillary dropout, as well as impaired glomerular filtration barrier function (26). In addition, MCP-1–mediated macrophage recruitment accelerates fibrovascular proliferation in the retina and tubulointerstitial fibrosis in the kidney (9).

Angiogenic pathways interact dynamically with inflammatory cascades. IL-6 and IL-8 enhance VEGF expression through STAT3 and MAPK activation, while VEGF further promotes vascular permeability and leukocyte recruitment, forming a self-amplifying inflammatory-angiogenic loop (27). Clinically, elevations in VEGF, TNF-α, IL-6, IL-8, MCP-1, and ICAM-1 in serum and vitreous correlate with DR progression and reduced renal function (28). In PDR, excessive VEGF drives pathological neovascularization, whereas in DKD, VEGF contributes to glomerular endothelial injury and proteinuria (29). TNF-α impairs vascular barrier integrity and contributes to capillary rarefaction in both tissues (30). IL-6, IL-8, and MCP-1 propagate chronic inflammation and oxidative injury, exacerbating structural damage (31).

Fibrosis-related mediators (e.g., TGF-β, CTGF) promote extracellular matrix deposition in late disease stages, contributing to fibrovascular proliferation in PDR and glomerulosclerosis/interstitial fibrosis in DKD (32). Therefore, VEGF-driven angiogenesis, TNF-α/IL-6-mediated inflammation, and TGF-β/CTGF-driven fibrosis collectively propel microvascular deterioration in both organs (33). A consolidated summary of the major cytokines implicated in DR and DKD—including their expression trends and biological functions—is presented in **Table 3**.

**Table 3:** Key cytokines in diabetic retinopathy and diabetic kidney disease: expression, function, and biomarker potential.

Cytokine	Expression and function in DR	Expression and function in DKD	Biomarker potential
VEGF	Significantly elevated in vitreous of PDR; increases vascular permeability	Mediates glomerular filtration barrier injury; promotes proteinuria	Predicts response to anti-VEGF therapy; vitreous and serum levels correlated
TNF-α	Promotes inflammatory cascades; disrupts blood–retinal barrier	Induces glomerular and tubulointerstitial inflammation, apoptosis, and fibrosis	Highly associated with DKD stage; identifies high-inflammation risk patients
IL-6	Elevated in both ocular and serum samples; promotes chronic inflammation and vascular permeability abnormalities	Involved in renal inflammation and fibrosis; positively correlates with UACR and eGFR decline	Reflects systemic inflammation; predicts combined DR+DKD progression
IL-8	Significantly elevated in vitreous of PDR	Associated with tubulointerstitial fibrosis; promotes inflammatory infiltration	Vitreous levels correlate with renal function; marker of inflammatory activity
MCP-1	Upregulated; recruits macrophages; correlates with retinal inflammation severity	Key chemokine in DKD; drives macrophage infiltration and renal fibrosis	Urinary MCP-1 is a DKD biomarker; reflects systemic inflammation
TGF-β	Elevated in early NPDR; regulates pericyte apoptosis	Key mediator of renal fibrosis; promotes ECM accumulation	Marker of tissue fibrosis progression; useful for evaluating therapeutic effect
CTGF	Involved in retinal fibrovascular membrane formation	Core mediator of DKD fibrosis; downstream of TGF-β signaling	Marker of renal fibrotic activity; target for anti-fibrotic therapy

The parallel elevation of these mediators in ocular and systemic circulation highlights that DR can serve as a “window” into systemic microvascular pathology. Cytokine profiling may facilitate DKD risk prediction and inform multitargeted therapeutic strategies integrating anti-angiogenic, anti-inflammatory, and anti-fibrotic modalities to preserve microvascular function in the eye and kidney.

### 4.3 Cytokine-focused therapeutic innovations in diabetic microvascular complication

Building on the shared pathogenic pathways linking the retina and kidney, elevated VEGF, TNF-α, and IL-6 levels in serum and vitreous closely correlate with DR progression and renal dysfunction, supporting the presence of a systemic inflammatory “eye–kidney axis” (34). Therefore, therapeutic strategies targeting cytokines should aim to modulate both local retinal inflammation and systemic inflammatory responses.

Anti-VEGF agents (e.g., ranibizumab) remain the standard of care for DME and PDR due to their ability to reduce vascular permeability and pathological neovascularization (35). However, although intravitreal anti-VEGF therapy can transiently decrease circulating VEGF levels, its effects on the chronic systemic inflammatory burden in DKD are limited and insufficient to prevent progressive renal microvascular damage (36).

Consequently, systemic interventions are increasingly being investigated for their potential to modulate inflammatory pathways relevant to both DR and DKD. Sodium–glucose cotransporter-2 inhibitors (e.g., empagliflozin) improve renal outcomes and simultaneously reduce levels of inflammatory cytokines such as TNF-α and IL-6, indirectly slowing DR progression (37). Likewise, GLP-1 receptor agonists (e.g., liraglutide) exhibit anti-inflammatory and antioxidant properties, enhance endothelial function, and confer cardiovascular and renal protection, suggesting additional benefits for retinal microvascular health (38). Moreover, renin–angiotensin–aldosterone system (RAAS) inhibitors (ACEIs/ARBs) attenuate TGF-β and CTGF activity and reduce renal fibrosis (39), which may extend to broader protection against retinal microvascular remodeling.

Recent advances in inflammation- and fibrosis-targeted therapies offer new opportunities for integrated management. Shared pathways such as NLRP3 inflammasome activation, IL-1β signaling, and NF-κB–mediated cytokine amplification contribute to endothelial injury in both organs. Emerging therapeutics—including NLRP3 inhibitors (e.g., MCC950) and IL-1β antagonists (e.g., canakinumab)—have shown strong anti-inflammatory and anti-fibrotic potential in preclinical and early-phase clinical studies, indicating promising dual-targeting strategies for microvascular complications (40).

Overall, cytokine-based therapeutic development in DR and DKD is transitioning from a predominantly local anti-VEGF approach toward comprehensive systemic inflammation modulation and multi-target intervention, with the goal of achieving precise and coordinated protection of both ocular and renal microvasculature.

## 5 Challenges and future directions

Despite advances in clarifying the cytokine-mediated link between DR and DKD, important challenges remain. Cytokine networks are highly interconnected and functionally redundant; thus, inhibition of a single mediator may not sufficiently suppress chronic microvascular inflammation. Combination therapies targeting multiple molecular pathways will likely be necessary to achieve optimal clinical benefits. Additionally, vitreous sampling is invasive and impractical for routine use, underscoring the need for reliable, minimally invasive biomarker platforms.

Future research should prioritize three major areas. First, multi-omics and systems biology approaches may reveal shared regulatory networks involving inflammation, angiogenesis, and fibrosis along the DR–DKD axis. Second, alternative sample sources—such as tears or aqueous humor—may provide more accessible biomarkers. Third, predictive models integrating imaging analytics, molecular signatures, and genetic determinants may enable more accurate risk stratification and early identification of patients at high risk of microvascular complications.

Personalized medicine will also play an essential role. Tailoring interventions to an individual’s cytokine profile, inflammatory burden, and genetic predisposition could enhance therapeutic efficacy while minimizing systemic adverse effects. Early intervention is equally critical, as modifying inflammatory responses during subclinical disease may more effectively delay progression of both DR and DKD.

This review has some limitations. Most included studies were cross-sectional, which restricts causal interpretation. Small sample sizes, single-center design, and variability in measurement methodologies may introduce heterogeneity. Moreover, inconsistent adjustment for confounders such as glycemic control and duration of diabetes limits the interpretability of clinical data. To confirm the central role of cytokines in the “eye–kidney microvascular inflammatory axis,” well-designed prospective multicenter studies are urgently required.

## 6 Conclusion

This systematic review highlights a consistent association between vitreous and serum levels of angiogenic and inflammatory cytokines in patients with DR and the progression of DKD, underscoring the pivotal role of the eye–kidney microvascular axis in the pathogenesis of diabetic microvascular complications. Future studies should focus on large-scale, prospective cohorts and leverage integrative approaches, including multi-omics and molecular imaging, to further elucidate the underlying mechanistic pathways of the eye–kidney axis and to evaluate its potential utility for early risk stratification and targeted therapeutic intervention.

## Data Availability

The original contributions presented in the study are included in the article/supplementary material. Further inquiries can be directed to the corresponding author/s.

## Glossary

ACEI: angiotensin-converting enzyme inhibitor
ACR: albumin-to-creatinine ratio
AGE: advanced glycation end product
ANGPTL2: angiopoietin-like protein 2
ANGPTL4: angiopoietin-like protein 4
ARB: angiotensin II receptor blocker
BUN: blood urea nitrogen
CTGF: connective tissue growth factor
DKD: diabetic kidney disease
DM: diabetes mellitus
DME: diabetic macular edema
DR: diabetic retinopathy
eGFR: estimated glomerular filtration rate
ELISA: enzyme-linked immunosorbent assay
FABP4: fatty acid-binding protein 4
GLP-1 RA: glucagon-like peptide-1 receptor agonist
HbA1c: hemoglobin A1c
ICAM-1: intercellular adhesion molecule-1
IL: interleukin
MCP-1: monocyte chemoattractant protein-1
NF-κB: nuclear factor kappa-light-chain-enhancer of activated B cells
NLRP3: NOD-like receptor family pyrin domain containing 3
NPDR: non-proliferative diabetic retinopathy
NOS: Newcastle–Ottawa Scale
PDR: proliferative diabetic retinopathy
PlGF: placental growth factor
PRISMA: Preferred Reporting Items for Systematic Reviews and Meta-Analyses
RAAS: renin–angiotensin–aldosterone system
RAGE: receptor for advanced glycation end products
ROS: reactive oxygen species
s(P)RR: soluble (pro)renin receptor
sCr: serum creatinine
sRAGE: soluble receptor for advanced glycation end products
STAT3: signal transducer and activator of transcription 3
T1DM: type 1 diabetes mellitus
T2DM: type 2 diabetes mellitus
TGF-β: transforming growth factor-β
TNF-α: tumor necrosis factor-α
UACR: urinary albumin-to-creatinine ratio
VEGF: vascular endothelial growth factor
